# The unfolded protein response alongside the diauxic shift of yeast cells and its involvement in mitochondria enlargement

**DOI:** 10.1038/s41598-019-49146-5

**Published:** 2019-09-04

**Authors:** Duc Minh Tran, Yuki Ishiwata-Kimata, Thanh Chi Mai, Minoru Kubo, Yukio Kimata

**Affiliations:** 1 Graduate School of Science and Technology, Nara Institute of Science and Technology, 8916-5 Takayama, Ikoma, Nara 630-0192 Japan; 2 Institute of Biotechnology, Vietnam Academy of Science and Technology, 18 Hoang Quoc Viet road, Cau Giay, Ha Noi Vietnam; 3 Institute for Research Initiatives, Nara Institute of Science and Technology, 8916-5 Takayama, Ikoma, Nara 630-0192 Japan; 4 Present Address: Graduate School of Science and Technology, Kumamoto University, 2-39-1 Kurokami, Chuo-ku, Kumamoto 860-8555 Japan

**Keywords:** Stress signalling, Fungal biology

## Abstract

Upon dysfunction of the endoplasmic reticulum (ER), eukaryotic cells evoke the unfolded protein response (UPR), which, in yeast *Saccharomyces cerevisaie* cells, is promoted by the ER-located transmembrane endoribonuclease Ire1. When activated, Ire1 splices and matures the *HAC*1 mRNA which encodes a transcription-factor protein that is responsible for the gene induction of the UPR. Here we propose that this signaling pathway is also used in cellular adaptation upon diauxic shift, in which cells shift from fermentative phase (fast growth) to mitochondrial respiration phase (slower growth). Splicing of the *HAC*1 mRNA was induced upon diauxic shift of cells cultured in glucose-based media or in cells transferred from glucose-based medium to non-fermentable glycerol-based medium. Activation of Ire1 in this situation was not due to ER accumulation of unfolded proteins, and was mediated by reactive oxygen species (ROS), which are byproducts of aerobic respiration. Here we also show that the UPR induced by diauxic shift causes enlargement of the mitochondria, and thus contributes to cellular growth under non-fermentative conditions, in addition to transcriptional induction of the canonical UPR target genes, which includes those encoding ER-located molecular chaperones and protein-folding enzymes.

## Introduction

The endoplasmic reticulum (ER) is a cellular compartment in which secretory and transmembrane proteins are folded. Membrane-lipid components are also mainly biosynthesized in the ER. Accumulation of unfolded proteins in the ER, which is tightly linked to dysfunction of the ER, is called ER stress, and triggers the unfolded protein response (UPR) in eukaryotic cells. Ire1, an ER-located type-I transmembrane endoribonuclease conserved in eukaryotic species, serves as an ER-stress sensor that triggers the UPR^1,2^. In ER-stressed *Saccharomyces cerevisiae* (hereafter simply called yeast) cells, the *HAC1* mRNA is spliced by activated Ire1 molecules and then translated into a transcription-factor protein, which functions in UPR transcriptional induction.

The molecular mechanism through which ER-accumulated unfolded proteins activate Ire1 is well documented. Under non-stress conditions, Ire1 is associated with the ER-located HSP70-family molecular chaperone BiP^3,4^. The presence of unfolded proteins causes dissociation of BiP from Ire1, which then self-associates (probably forming a dimer) although this is not the fully activated state^5,6^. According to previous reports by us and others^7–9^, the self-associated forms of yeast Ire1 and IRE1α (the major version of the mammalian Ire1 paralogues) directly capture unfolded proteins accumulated in the ER for their high-order oligomerization and full activation.

It should also be noted that the yeast Ire1-*HAC1* pathway of the UPR transcriptionally induces a number of genes which not only include ER-located molecular chaperones and protein-folding enzymes but also enzymes involved in generation of membrane-lipid components and in elimination of reactive oxygen species (ROS)^10,11^. Moreover, membrane-lipid aberrancy is likely to activate Ire1 independently of accumulation of unfolded proteins in the ER^12–14^. The UPR thus seems to have functions other than in coping with ER accumulation of unfolded proteins. We have therefore searched for new scenarios in which the UPR functions in yeast cells.

When inoculated into a medium rich in fermentable sugar, such as glucose, yeast cells are fueled mainly by the fermentation for rapid growth. Cell growth is then retarded upon exhaustion of the fermentable sugar, as cells utilize the fermentation products as carbon and energy sources via respiration. This switch from anaerobic growth to aerobic respiration is called the diauxic shift, and is accompanied by a drastic change in the gene expression profile and by massive expansion of the mitochondria^15^. Here we report UPR induction in yeast cells upon diauxic shift even in the absence of external stressing stimuli. Intriguingly, in this case, Ire1 is likely to be activated not by ER-accumulated unfolded proteins but by ROS. We also note the involvement of this phenomenon in expansion of the mitochondria.

## Results

### The Ire1-*HAC1* signaling pathway of the UPR is activated upon diauxic shift in yeast cells

**Figure 1:**
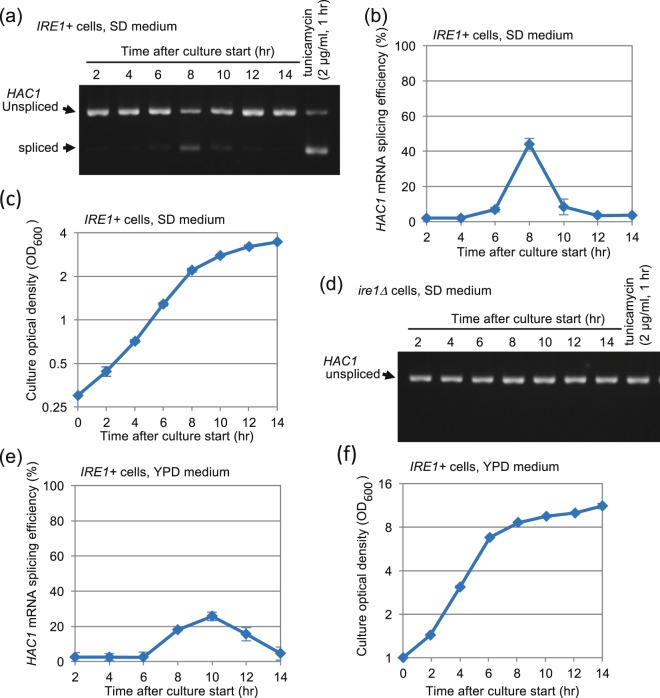
*HAC1*-mRNA splicing is transiently induced upon diauxic shift of yeast cells. (**a**) After being cultured for the indicated durations in SD medium or stressed by the canonical ER stressor tunicamycin, *IRE1*+ cells were analyzed by RT-PCR amplification of the *HAC1* transcripts (See Fig. S11 for the uncropped gel image). (**b**) The same experiment as shown in panel A was performed using three independent clones of *IRE1*+ cells to obtain quantified data. (**c**) Optical density of the cultures analyzed in panel B. (**d**) The same experiment as described in panel A was performed using *ire1Δ* cells (See Fig. S11 for the uncropped gel image). (**e**,**f**) *IRE1*+ cells were cultured in YPD, and analyzed for *HAC1*-mRNA splicing and culture density.

This research was initiated by our finding that a considerable but transient UPR is observed upon long-time culture of yeast cells in standard synthetic dextrose (SD) medium. Unless otherwise noted, throughout this study, yeast strains were incubated overnight in SD medium, and then the resulting pre-cultures were diluted in the same medium (to produce an OD_600_ of 0.30), cultured further and harvested after a time-course. Figure 1a,b demonstrate a transient induction of *HAC1*-mRNA splicing in *IRE1*+ cells cultured using this procedure. A more detailed time-course profile of the transient induction of *HAC1* mRNA splicing is shown in Fig. S1. We deduce that this phenomenon is concurrent with the diauxic shift, since after this time point (8-hr after culture start), cells grew slowly (Fig. 1c). Table S1 indicates slopes of the lines of Fig. 1c and of the other growth charts shown later.

**Figure 2:**
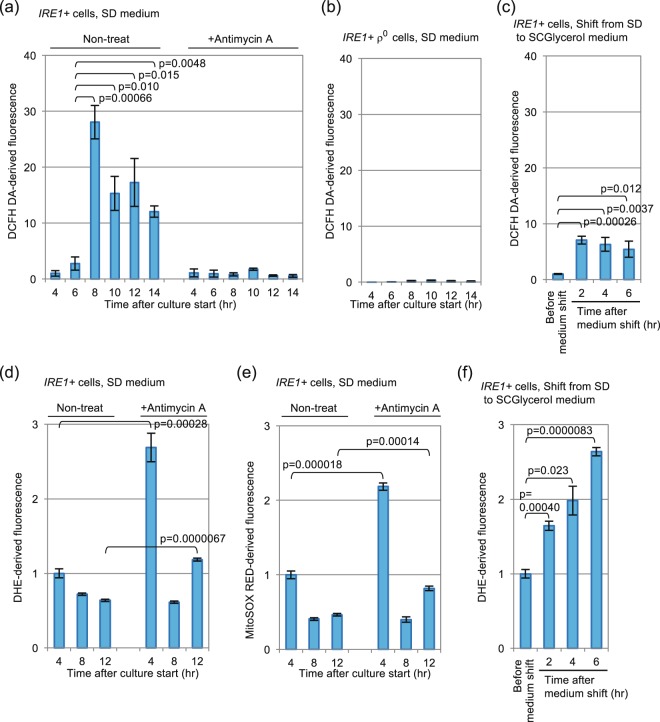
Intracellular ROS level under the culturing conditions employed in this study. After being cultured in SD medium (**a**,**b**,**d**,**e**) or shifted from SD to SCGlycerol medium (**c**,**f**) for the indicated durations, *IRE1*+ cells or ρ^0^ *IRE1*+ cells were stained with DCFH-DA, DHE or MitoSOX for 30 min, and were analyzed by flowcytometry. In panel a, d and e, antimycin A (20 µM final concentration) was added into medium concomitantly with culture start. In all panels, all fluorescent values are normalized against that of the fermentatively growing *IRE1*+ cells (4-hr after culture start, Non-treated), which is set at 1.00.

In order to confirm that cells reached the diauxic-shift phase 8-hr after culture start, we checked the level of intracellular ROS, which are byproducts of aerobic respiration. In the experiment shown in Fig. 2a (Non-treat), we stained cells with cell permeant reagent 2′-7′ dichlorodihydrofluorescin diacetate (DCFH-DA), which is converted to a fluorescent form by peroxides^16^, and found a drastic increment of the cellular ROS level 8-hr after culture start (and later time points). However, such was not observed when we used the superoxide-anion indicator dihydroethidium (DHE) or its mitochondria-targeted derivative MitoSOX (Fig. 2d,e; Non-treat).

As well as in the case of canonical ER-stressing stimuli, the *HAC1*-mRNA splicing shown in this study is caused by Ire1, since no *HAC1*-mRNA splicing was observed in cells lacking the *IRE1* gene (the *ire1Δ* mutation) even when they reached diauxic shift (Fig. 1d, see Fig. S3a for growth profile of *ire1Δ* cells). Moreover, as expected, the K1058A endoribonuclease-deficient mutant of Ire1 failed to splice the *HAC1* mRNA when cells reached to the diauxic shift condition (Fig. S2). As shown in Fig. S4, another *IRE1*+ strain that is not congenic with the strains used throughout this study exhibited similar transient *HAC1*-mRNA splicing upon long-term culture. Western-blot detection of the hemagglutinin (HA)-epitope tagged Ire1 indicates that the cellular level of Ire1 is almost unaffected during the culturing procedure employed in this study (Fig. S5). Transient *HAC1*-mRNA splicing at the end of the exponential cell growth was also observed when cells were grown in the rich yeast extract-peptone-dextrose (YPD) medium (Fig. 1e,f).

We then compared growth of *IRE1*+ cells with that of *ire1Δ* cells in the experiment shown in Fig. S3a, the result of which is statistically analyzed in Table S2, and found that the *ire1Δ* mutation retards cellular growth alongside diauxic shift (8-hr after culture start). As mentioned later in the last sub-section of the Results section, it should be also noted that, even in later time points, the *ire1Δ* mutation conferred negative effects on cells, which however was not expressed as an apparent growth retardation (Table S2) probably because of the limitation of experimental techniques. We think that, on the post diauxic-shift phase, cellular grow rate is too slow to exhibit a biological variation that dominates over technical variations. Fig. S3b (and Table S2) indicates that, as expected, the *hac1Δ* mutation caused similar growth retardation as the *ire1Δ* mutation.

### ER protein folding is not impaired upon diauxic shift of yeast cells

In general, Ire1 is thought to exert its potent *HAC1*-mRNA splicing activity through sensing ER accumulation of unfolded proteins^17^. The obvious question is therefore whether protein folding in the ER is affected upon diauxic shift of yeast cells?

In the experiment shown in Fig. S6a, we performed a BiP sedimentation assay^12,18,19^, via which we can estimate production of ER protein aggregates that incorporate BiP. In this assay, cell lysates were fractionated by centrifugation before being analyzed by anti-BiP Western blotting. A disulfide-bond reducing agent dithiothreitol (DTT) or an N-glycosylation inhibitor tunicamycin are known to impair protein folding in the ER and to induce potent ER stress. While treatment of cells with DTT or tunicamycin considerably increased the BiP level in the pellet fraction, the same was not observed with diauxic shift.

We next employed yeast cells expressing eroGFP, which is an ER-located version of GFP that changes its excitation spectrum in a way that is dependent on intramolecular disulfide-bond formation^20^. As shown in Fig. S6b, DTT treatment of cells, but not diauxic shift caused a considerable change in the excitation profile of eroGFP, suggesting that diauxic shift does not significantly disturb the disulfide bond-forming ability of the yeast ER. We did not employ tunicamycin as a positive control for this assay, because it is unlikely that tunicamycin induces ER stress through impairment of the disulfide-bond formation.

**Figure 3:**
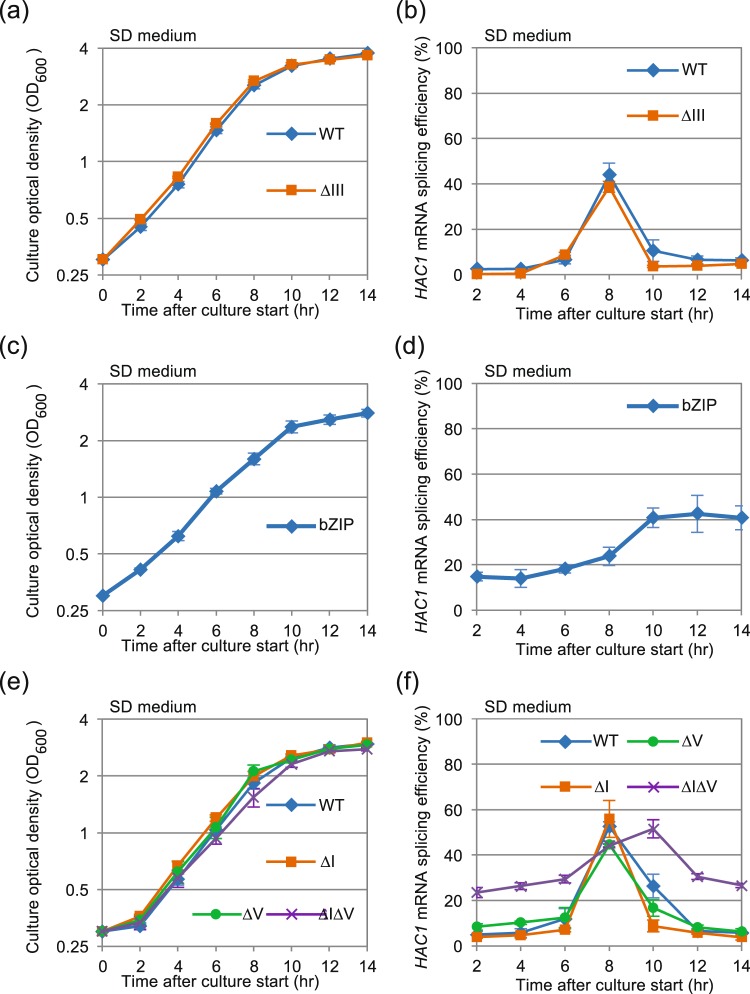
Effect of the luminal-domain mutations of Ire1 on the *HAC1* mRNA-splicing profile during long-time culture. Cells carrying the indicated mutations of the *IRE1* gene were analyzed as per Fig. 1b,c.

In order to provide further evidence for our idea that diauxic shift activates Ire1 without ER accumulation of unfolded proteins, we employed a partial deletion mutant of Ire1, namely ΔIII Ire1 (Fig. S7), which is impaired in sensing of unfolded proteins^7,12^. Importantly, this deletion mutant is insufficiently activated by unfolded proteins which accumulate in the ER^7^, but can trigger the UPR to an almost equal extent as wild-type Ire1 in response to certain stressing stimuli^12^. Figure 3a,b indicate that cells carrying the ΔIII Ire1 mutation exhibit an almost identical *HAC1* mRNA-splicing profile as wild-type Ire1 cells upon diauxic shift.

### Regulatory roles of domains of Ire1 in transient activation upon diauxic shift

In order to investigate the regulatory mechanism of Ire1 upon diauxic shift, we further employed yeast cells carrying luminal-domain mutants of Ire1, which are illustrated in Fig. S7. The bZIP mutation of Ire1 is a replacement of its full-length luminal domain with a dimer-forming basic leucine-zipper (bZIP) peptide that is derived from the nuclear transcription factor protein Gcn4^12^. As shown in Fig. 3c,d and as reported previously^12^, bZIP-Ire1 cells exhibit considerable *HAC1*-mRNA splicing even when exponentially and fermentatively growing, since bZIP-Ire1 is constitutively self-associated through its bZIP domain. Importantly, splicing of the *HAC1* mRNA in bZIP-Ire1 cell was further stimulated upon diauxic shift, and, unlike the case for wild-type *IRE1* cells, was not attenuated in the post diauxic-shift phase (Fig. 3c,d). We thus speculate that an activation signal functions at the cytosolic (or transmembrane) domain of Ire1 for the UPR evocation upon diauxic shift. Nevertheless, the luminal domain of Ire1 also seems to contribute to the regulation of Ire1, since wild-type Ire1 but not bZIP-Ire1 was downregulated after diauxic shift.

According to previous observations by us and others, the luminal domain of Ire1 carries two subregions, namely the N-terminal Subregion I and the juxtamembrane-positioned Subregion V (Fig. S7), which contribute to downregulation of Ire1^5,6,21^. We therefore examined the contribution of these subregions to regulation of Ire1 in the transient evocation of UPR upon diauxic shift. Consistent with our previous observation^22^, cells carrying deletions of both Subregions I and V (the ΔIΔV mutation) exhibited substantial *HAC1* mRNA splicing even when growing exponentially and fermentatively (Fig. 3e,f). Here we note transient stimulation of the *HAC1* mRNA splicing upon diauxic shift in ΔIΔV Ire1 cells, which implies that neither Subregion I nor V plays an important role in regulation of Ire1 in this case.

### Involvement of mitochondrial respiration and ROS in Ire1 activation upon diauxic shift

**Figure 4:**
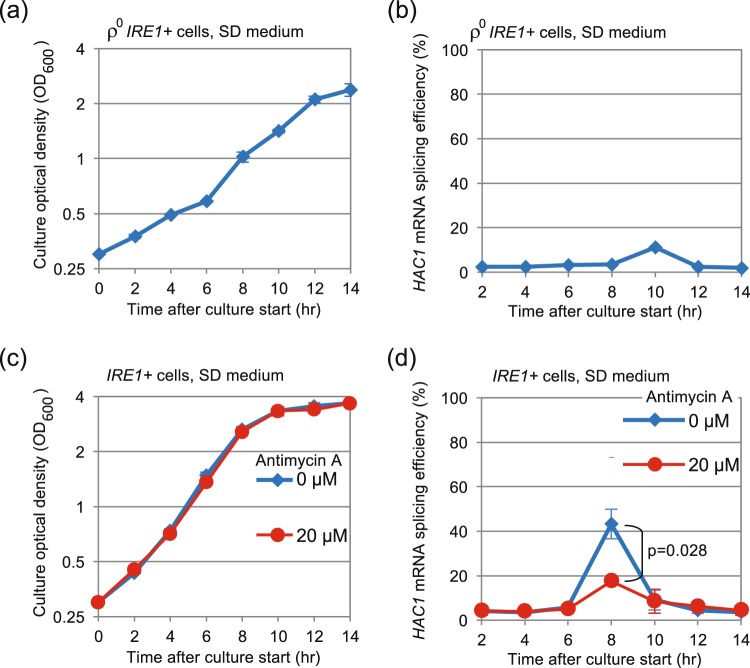
The ρ^0^ mutation and antimycin A impair the *HAC1*-mRNA splicing alongside long-time culturing. (**a**,**b**) The same experiments as described in Fig. 1b,c were performed using ρ^0^ *IRE1*+ cells. (**c**,**d**) Antimycin A was added into culture at time 0, and the same experiments as described in Fig. 1b,c were performed.

We next checked the relationship between the transient evocation of the UPR and mitochondrial aerobic respiration, which is known to be stimulated upon diauxic shift. The ρ° mutation of yeast cells is a loss of the mitochondrial genome and is known to abolish aerobic respiration. Figure 4a,b show that the *HAC1*-mRNA splicing observed at the end of the exponential and fast cell growth phase was only faintly detectable in ρ° mutant cells (compare to Fig. 1b). According to our data shown in Fig. 2b,e, ROS level in ρ^0^ cells was not increased even when they reached a growth-retardation phase (Fig. 2b).

In the experiment shown in Fig. 4c,d, *IRE1*+ cells were cultured in the presence of a respiratory inhibitor antimycin A (20 µM), which did not affect cellular growth, but did impair the *HAC1*-mRNA splicing upon diauxic shift and lower the cellular ROS level that was monitored by DCFH-DA (Fig. 2a).

Meanwhile, in general, antimycin A is known to induce ROS production under various situations^16,23^. Consistently, in our hands, all of the ROS indicators used in this study showed an antimycin A-induced increment of the ROS level in PBS-suspended cells (Fig. S8). Moreover, the DHE-derived or MitoSOX-derived fluorescent signals from cells being cultured in SD medium containing antimycin A was stronger than those from cells being cultured without antimycin A (Fig. 2d,e).

**Figure 5:**
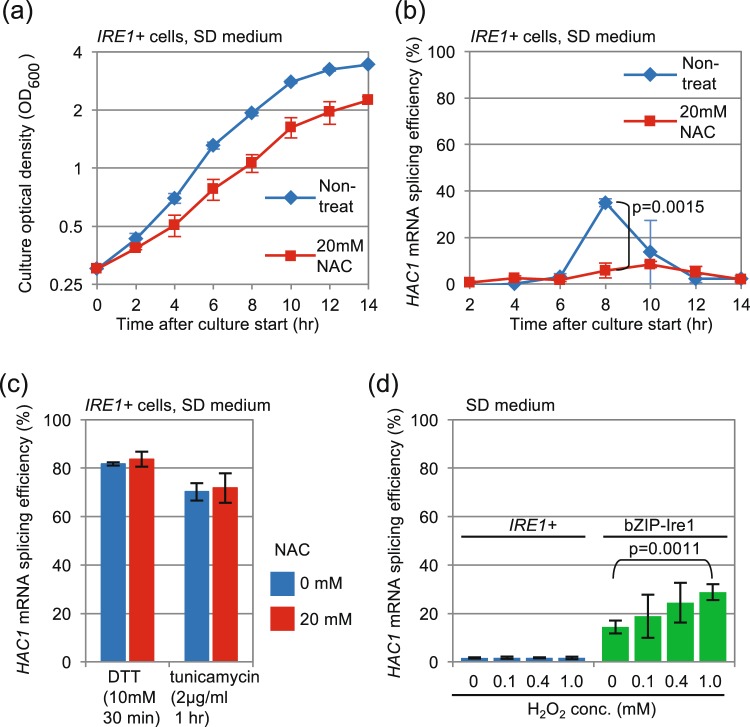
ROS contribute to but are insufficient for Ire1 activation upon diauxic shift. (**a**,**b**) *IRE1*+ cells were cultured in the presence or absence of 20 mM NAC and monitored for growth and *HAC1*-mRNA splicing. (**c**) *IRE1*+ cells were treated with ER-stressing chemicals as indicated and checked for *HAC1*-mRNA splicing. NAC and ER stressors were simultaneously added into cultures. (**d**) *IRE1*+ cells or cells carrying bZIP-Ire1 were treated with the indicated concentrations of hydrogen peroxide for 30 min or remained unstressed, and checked for *HAC1*-mRNA splicing.

Since we hypothesized that ROS may contribute to the induction of *HAC1*-mRNA splicing caused by diauxic shift, we verified this idea through addition of the ROS scavenger N-acetyl-L-cysteine (NAC) into cultures of *IRE1*+ cells, which slowed cellular growth (Fig. 5a). Importantly and as expected, NAC suppressed the *HAC1*-mRNA splicing upon diauxic shift (Fig. 5b). It also should be noted that conventional ER stressors, DTT and tunicamycin, potently induced *HAC1*-mRNA splicing both in the presence and absence of NAC (Fig. 5c).

In the experiment shown in Fig. 5d, exponentially growing *IRE1*+ cells were treated with hydrogen peroxide, which did not induce the *HAC1*-mRNA splicing. However, hydrogen peroxide stimulated *HAC1*-mRNA splicing in cells carrying the bZIP mutant version of Ire1. We thus assume that the transmembrane or the cytosolic domain of Ire1 is influenced by ROS, which contributes to but is not sufficient for UPR evocation.

**Figure 6:**
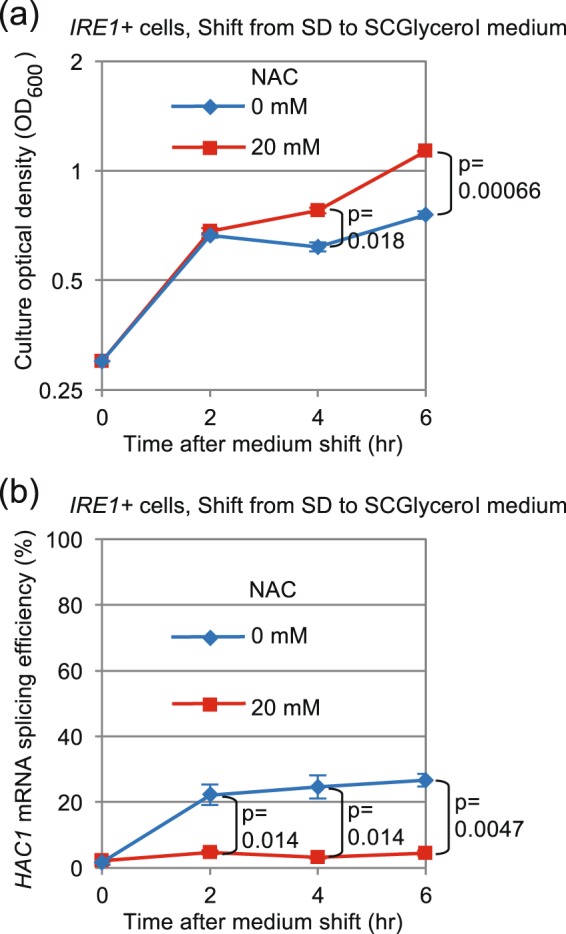
Sustained *HAC1*-mRNA splicing is induced in cells shifted from a fermentable to a non-fermentable medium. *IRE1*+ cells exponentially growing in SD medium were transferred to SCGlycerol medium at time 0, further incubated for the indicated durations and checked for culture density (**a**) and *HAC1*-mRNA splicing (**b**). For “20 mM NAC” samples, NAC was added into culture at time 0.

The experiments shown in Fig. 6 support our proposal that *HAC1*-mRNA splicing is tightly linked to aerobic respiration and ROS production. Yeast cells are known to be predominantly fueled by aerobic respiration in a non-fermentable glycerol-based medium. When wild-type cells fermentatively growing in SD medium were transferred to SCGlycerol medium, they exhibited considerable and continuing levels of *HAC1*-mRNA splicing (Fig. 6b). Unlike the long-time culturing in SD medium, this medium shift caused ROS production that was observable not only by DCFH-DA but also by DHE (Fig. 2c,f). Intriguingly, NAC did not inhibit but did support cellular growth after shifting to SCGlycerol (Fig. 6a). We thus propose that, under this condition, ROS stress is too severe for cells to grow and to attenuate Ire1 activation. As expected, NAC abolished *HAC1*-mRNA splicing in this culture condition (Fig. 6b).

### Ire1-dependent expansion of the mitochondria alongside diauxic shift of yeast cells

**Figure 7:**
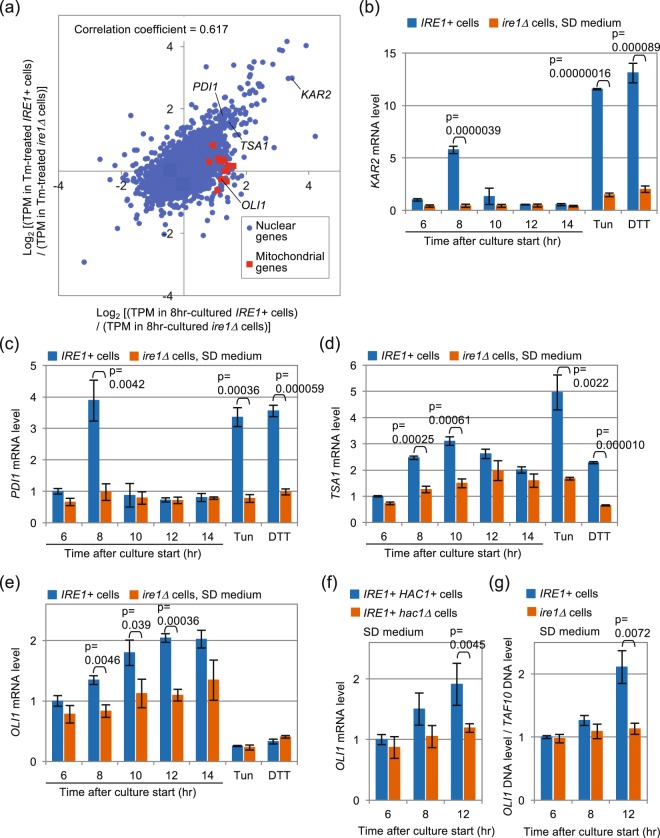
Ire1-dependent change of gene-expression profile under different conditions. (**a**) *IRE1*+ cells (two clones) and *ire1Δ* cells (two clones) upon diauxic shift (8 hr-culturing in SD medium) or under the canonical ER stress condition (2 µg/ml tunicamycin of 1 hr) were subjected to Illumina-based RNA sequencing and gene-expression profiling. The resulting TPM (transcripts per million) values of the resulting clones were used to calculate the x-axis and y-axis values of each gene. In this scatter plot, each dot represents one gene (5854 genes in total). Genes with TPM values of less than 1.00 under any conditions were not plotted. The *ULI1* gene is plotted out of the graph area and is not shown here. (**b**–**e**) *IRE1*+ cells and *ire1Δ* cells were cultured in SD medium with or without the indicated external stress, and checked for the abundance of mRNAs of the indicated genes using qRT-PCR. (**f**) The same experiment as described in panel e was performed using the indicated strains. (**g**) Total DNA samples were analyzed by qPCR using *OLI1*-gene (mitochondria genome) specific and *TAF10*-gene (nuclear genome) specific primers. Expression levels of each gene (or ratios of *OLI1* DNA level to *TAF10* DNA level) are normalized against that of *IRE1*+ cells at time 6 hours, which is set at 1.00.

According to Thibault *et al*.^24^, the downstream target genes of the UPR in yeast cells vary dependent on the type of stressing stimuli. We thus compared the Ire1-dependent transcriptional changes caused by diauxic shift with those caused by canonical ER-stressing stimulus. In the experiments shown in Fig. 7a and Table S3, we performed RNA-sequencing-based transcriptome analysis of *IRE1*+ cells and *ire1Δ* cells stressed by tunicamycin or during diauxic shift. Figure 7a indicates a positive correlation between the *IRE1*-dependent transcriptome change in tunicamycin-stressed cells (Y-axis) and that in cells undergoing diauxic shift (X-axis) with the correlation coefficient of 0.617. Indeed, expression of some previously described UPR target genes, for example, *KAR2*, *PDI1* and *TSA1*^10,11^ were induced dependent on Ire1 in response to both canonical ER stressing stimuli (DTT or tunicamycin) and diauxic shift (Fig. 7b–d). In contrast, expression of genes carried on the mitochondrial genome upon diauxic shift but not upon tunicamycin stress was highly dependent on Ire1 (Fig. 7a). Time-course expression monitoring of *OLI1*, which serves as an example of the mitochondrial encoded genes, indicated that its upregulation upon diauxic shift is impaired in the *ire1Δ* mutant (Fig. 7e). Meanwhile, the mRNA level of *OLI1* was considerably suppressed by canonical ER stressing stimuli independently of the *IRE1* gene (Fig. 7e). As expected, the *hac1Δ* mutation also impaired *OLI1* induction upon diauxic shift (Fig. 7f). Figure 7g shows that not only the RNA level but also the DNA level of *OLI1* was induced in an Ire1 dependent manner upon diauxic shift. We thus propose that, at least partly, Ire1 increases the mitochondrial RNA abundance through an increase in mitochondrial DNA abundance. In the experiments shown in Fig. S9, we observed similar changes in the cellular RNA and DNA abundance of other mitochondrial genes between before and after diauxic shift.

**Figure 8:**
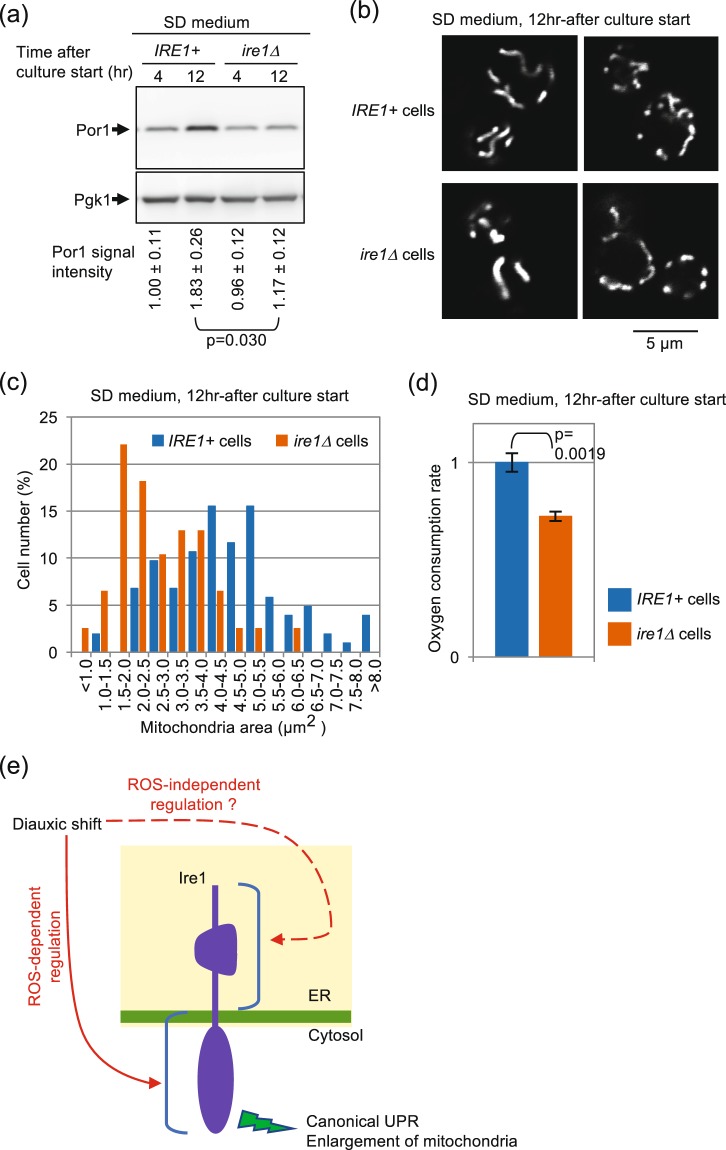
Ire1-dependent enhancement of respiration and mitochondria size after diauxic shift. *IRE1*+ cells and *ire1Δ* cells were cultured in SD medium for the indicated durations. (**a**) Cell lysates (equivalent to 0.22 OD_600_ cells) were analyzed by anti-Por1 Western blotting. Anti-Pgk1 Western blot serves as a loading control. See Fig. S12 for the uncropped blot image. (**b**,**c**) Cells producing mitochondria-located GFP were optically sectioned by a confocal microscope and monitored for fluorescing area. Note that size distribution of *IRE1*+ cells and that of *ire1Δ* cells are statistically different (p = 3.0 × 10^13^). (**d**) Oxygen consumption rates were assayed as described in the Materials and Methods section. (**e**) Novel activation mode and role of Ire1 presented in this study is illustrated.

We then compared the mitochondria-related cellular status of *IRE1*+ cells and *ire1Δ* cells. Figure 8a shows the cellular level of the mitochondria marker protein Por1, which was induced alongside diauxic shift in *IRE1*+ cells, but poorly in *ire1Δ* cells. According to the data from the transcriptome analysis shown in Table S3, the difference in the cellular Por1 protein level in *IRE1*+ and *ire1Δ* cells does not seem to be due to the level of transcription of the *POR1* gene. In the experiment shown in Fig. 8b,c, mitochondria in *IRE1*+ and *ire1Δ* cells in the post-diauxic-shift phase were visualized by mitochondria-located GFP. While mitochondria exhibited a tubular-like shape in both types of cells, a quantitative image analysis indicated that mitochondria are less expanded in *ire1Δ* cells. Supporting these insights, oxygen uptake in the post-diauxic-shift phase was lowered by the *ire1Δ* mutation (Fig. 8d). Meanwhile, these differences between *IRE1*+ cells and *ire1Δ* cells were not observed when we analyzed 8 hr-cultured samples (Fig. S10).

## Discussion

As the name denotes, the UPR has been believed to be a cellular response against accumulation of unfolded proteins in the ER lumen. Indeed, it is highly likely that the luminal domain of Ire1 has the ability to directly capture unfolded proteins, which leads to activation of Ire1 and evocation of the UPR^7–9,12,25^. However, in the present study, we cultured yeast cells in the two most commonly used media, YPD and SD, for long duration and noticed that the UPR is transiently induced upon diauxic shift (Figs 1, S1 and S4). Intriguingly, this phenomenon is unlikely to accompany ER accumulation of unfolded proteins, since wild-type Ire1 and the ΔIII mutant of Ire1 were equally activated (Fig. 2a,b). Moreover, the protein-folding status of the ER does not seem to be harmed upon diauxic shift (Fig. S6).

Probably because of weak but considerable UPR induction form the beginning of culture (Fig. 3d,f), cells producing bZIP Ire1 or ΔIΔV Ire1 grew slightly slower than the others and seemed to reach diauxic shift approximately 10-hr after the culture start (Fig. 3c,e and Table S1). Accordingly, the time points in which the *HAC1*-mRNA splicing is strongly induced were delayed in these cells (Fig. 3d,f). These observations support our idea that the potent induction of the *HAC1*-mRNA splicing upon long-time culturing is tightly linked to diauxic shift.

The transient induction of the UPR was only weakly observed in ρ° cells or in wild-type cells cultured with antimycin A (Fig. 4), and thus appears to be tightly linked to mitochondrial respiration. Consistent with this idea, the UPR was quickly induced when cells were transferred to non-fermentable glycerol-based medium (Fig. 6). This observation argues against the idea that the activation of Ire1 shown in Fig. 1 may be caused by exhaustion of trace nutrient components from medium along long-term culturing. Because NAC considerably impaired the UPR induction under these conditions (Figs 5b and 6b), ROS possibly serve as mediators for UPR induction resulting from aerobic respiration. Given the susceptibility of bZIP-Ire1 to hydrogen peroxide (Fig. 5d), we propose that ROS contribute to activation of Ire1 through directly or indirectly acting on its cytosolic or transmembrane domain. In contrast, unlike in the case of bZIP-Ire1 cells, hydrogen peroxide did not induce the UPR in *IRE1*+ cells (Fig. 5d). Moreover, the UPR upon diauxic shift was transient in *IRE1*+ cells (Fig. 1), while cellular level of ROS was decreased only modestly after diauxic shift (Fig. 2a). These observations support our idea that ROS contribute to but are not sufficient for UPR induction. It should be also noted that bZIP Ire1 was continuingly activated after diauxic shift (Fig. 3c,d).

As described above, DCFH-DA and DHE (and its mitochondria-targeted derivative MitoSOX) are thought to detect respectively peroxides and superoxide^16^. Based on the ROS-indicator fluorescent profiles shown in Fig. 2, we thus think that the *HAC1*-mRNA splicing shown in this study is tightly linked to the cellular level of peroxides but not to that of superoxide. It should be also noted that, in our experimental conditions, the peroxide and the superoxide levels are often unsynchronized. Along diauxic shift, the cellular level of peroxides was drastically increased, while that of superoxide seemed to be rather decreased in non-treated cells (Fig. 2a,d,e). Moreover, ainitmycin A increased superoxide level but drastically suppressed peroxide level in cells being cultured in SD medium (Fig. 2a,d,e; compare the antimycin A-treated samples to the non-treated samples). However, peroxide level, as well as superoxide level, was increased by antimycin A in cells suspended in PBS (Fig. S8). The molecular basis underlying these issues should be addressed in future.

As a possible explanation for these findings, we propose a hypothetical scenario which is illustrated in Fig. 8e. Diauxic shift influences Ire1 in two different ways, both of which are required for potent activation of Ire1. One is via ROS, which directly or indirectly works on the cytosolic domain (or the transmembrane domain) of Ire1. The other signal is still obscure and works via the luminal domain of Ire1. Because of the absence of the latter signal, wild-type Ire1 is not activated by ROS only. Moreover, because the latter signal is quickly attenuated, activation of wild-type Ire1 upon diauxic shift is only transient.

We previously reported that Subregion V serves as the BiP-binding site and negatively regulates Ire1^5^. Furthermore, Subregion I is likely to be intramolecularly associated with the cLD of Ire1 and inhibit its self-association under non-stress conditions^22^. According to our observations shown in Fig. 3e,f, as well as *IRE1*+ cells, cells carrying the ΔIΔV version of Ire1 evoked the UPR transiently upon diauxic shift. We thus speculate that neither Subregion I nor V plays a central role in the transient UPR induction and attenuation observed in this study.

Here we also show that Ire1-dependent *HAC1* splicing upon diauxic shift contributes to growth of yeast cells (Fig. S3 and Table S2). As is the case with canonical ER stressing stimuli, Ire1 controls expression of a number of genes (Fig. 7a), including the peroxiredoxin gene Tsa1 (Fig. 7d), upon diauxic shift. Thus, one of the physiological functions of the UPR induction upon diauxic shift may be to cope with ROS, which are byproducts of respiration. In addition, we also found Ire1-dependent increase of the mRNA level of mitochondrial genes upon and after diauxic shift (Figs 7a,e and S9). This phenomenon does not seem to be solely due to transcriptional induction of the mitochondrial genes, since the cellular level of the mitochondrial DNA was also increased under this condition (Figs 7g and S9). In contrast, canonical ER-stressing stimuli repressed the *OLI1* expression level independently of Ire1 (Fig. 7e). This observation argues that an outcome of the Ire1 activation upon diauxic shift differs from that caused by canonical ER-stressing stimuli, implying complexity in the intercellular signaling pathways that interact with the Ire1/*HAC1*-dependent UPR signaling pathway. Moreover, our findings shown in Fig. 8 indicate Ire1-dependent enhancement of size and function of the mitochondria after diauxic shift.

As shown in Figs 1 and 7, the time profile of the *HAC1*-mRNA splicing efficiency seems to be well synchronized with that of the *KAR2* or *PDI1*-gene expression, as both showed sharp peaks upon diauxic shift (8-hr after culture start). This observation is consistent with a previous report which argues that the Hac1 protein directly and positively acts to the promoter regions of *KAR2* and *PDI1*^26^.

Meanwhile, although also dependent on Ire1, the increment of the *OLI1* mRNA abundances was only faint upon diauxic shift (8 hr-after culture start) and was further enhanced on later time points. In agreement with this observation, the Ire1-dependent mitochondria expansion and respiration enhancement were observed after diauxic shift (12 hr-after culture start; Fig. 8) but not upon diauxic shift (8 hr-after culture start; Fig. S10). We thus think that these events are indirect outcomes that occur after activation of the Ire1-*HAC1* pathway.

A possible molecular basis underlying dependency of mitochondria expansion on the Ire1-*HAC1* pathway is offered by the fact that the UPR induces a wide variety of genes including those encoding enzymes for membrane-lipid biogenesis^10,11^, which would be required for expansion of the mitochondria, a membrane-bound cellular compartment. The contribution of ER-to-mitochondria lipid transport to maintenance of mitochondria homeostasis and integrity is reviewed in Scharwey *et al*.^27^. Moreover, Isc1, a sphingolipid biosynthetic-pathway enzyme, is reported to be required for enhancement of mitochondrial functions upon diauxic shift^28^.

As reviewed in Soontorngun^29^, various transcription factors and intracellular signaling pathways are complicatedly involved in the progression of diauxic shift in yeast cells. In conclusion of our present study, we describe that, in addition to them, the Ire1-*HAC1* pathway of the UPR contributes to the change of cellular status upon diauxic shift. As illustrated in Fig. 8e, ROS serve as possible mediators for this phenomenon and appear to act on the cytosolic domain of Ire1, although this is not sufficient for activation of Ire1. Evocation of the UPR under these conditions leads to expansion of the mitochondria and to stimulation of respiration, as well as to the traditionally known transcriptome change observed upon canonical ER stress. ROS production is thus likely to be both the cause and the result of the UPR induction upon diauxic shift. In other words, the phenomena observed here may constitute a positive feedback loop mediated by ROS (Fig. 8e). Taken together, we propose a new mode of action of the Ire1-*HAC1* signaling pathway of the UPR, which argues for a novel aspect of intracellular communication between two different organelles, the ER and the mitochondria in yeast cells.

## Materials and Methods

### Yeast culturing

Unless otherwise noted, yeast cells were cultured in SD medium containing 2% glucose, 0.66% yeast nitrogen base without amino acids (YNB w/o A.A.; Difco) and appropriate auxotrophic requirements. YPD medium contains 1% yeast extract (Bacto), 2% Peptone (Bacto) and 2% glucose. To support cellular growth in synthetic glycerol medium containing 5% glycerol and 0.66% YNB w/o A.A., we supplemented it with the standard histidine-dropout complete pre-mix, which is composed of 2.0 g each of 18 proteinogenic amino-acid powders (all standard proteinogenic amino acids excluding leucine and histidine), 10.0 g of leucine, 0.5 g of adenine, 2.0 g of uracil and 2.0 g of *p*-aminobenzoic acid, at a final concentration of 2.0 g/l (SCglycerol).

Unless otherwise noted, throughout this study, cells were shaken overnight at 30 °C in SD medium, and the resulting pre-cultures were diluted in the same medium (setting OD_600_ of 0.30) and shaken aerobically at 30 °C. In the experiments shown in Fig. 1e,f, overnight YPD pre-cultures were diluted 1 in 10 with YPD medium for further culturing at 30 °C. In the experiment shown in Fig. 6, cells in the exponential and fermentative growing phase in SD medium were washed with SCGlycerol medium for three times by repeated centrifugation and resuspension before being cultured in SCGlycerol medium. A spectrophotometer SmartSpec 3000 (BioRad) was used to measure optical density of cultures at 600 nm.

### Yeast strains and plasmids

Unless otherwise noted, we employed strains derived from W303-ire1Δ (*MAT****a**** ura*3-*1 trp1*-*1 leu2*-*3*,112 *his3*-11,15 *ADE2 ire1::TRP1 can1*-100; ref.^30^) throughout this study. Another strain KMY1005 (*MATα leu2*-3,112 *ura3*-52 *his3Δ*200 *trp1Δ*901 *lys2*-801; ref.^26^) was employed in the experiment shown in Fig. S4. KMY1516 (*MATα LEU2::UPRE*-*GFP LYS2::UPRE*-*lacZ ura3*-52 *his3Δ*200 *trp1Δ*901 *ire1::TRP1*; ref.^5^) is a derivative of KMY1005 and was used in the experiments shown in Figs 3c,d and 5d. To introduce the ρ° mutation, cells were cultured overnight in YPD medium containing 10 µg/ml ethidium bromide, and clones that cannot grow on glycerol-based medium were selected.

To generate *IRE1*+ cells, W303-ire1Δ were transformed with pRS313-IRE1^5^, which is a centromeric plasmid carrying the *IRE1* gene (the coding sequence plus the 5′- and 3′-untranslated regions). A centromeric plasmid that was used for expression of the C-terminally HA-tagged version of Ire1 (Ire1-HA), pRS315-IRE1-HA, is also referred to in our previous publication^5^. As described previously^5^, DNA fragments corresponding to the *IRE1*-luminal domain carrying partial deletion mutations were created through overlap PCR amplification from pRS313-IRE1 using primer sets listed in Table S4 and Takara pyrobest DNA polymerase. Through the standard Li-acetate transformation technique^31^, KMY1516 was transformed with a mixture of 500 ng of the resulting DNA fragment and 10 ng of SalI/XbaI-digested pRS313-IRE1, which were fused via *in vivo* homologous recombination to yield a circular pRS313-IRE1 DNA carrying a partial deletion mutation in yeast cells. A similar strategy was employed to create KMY1516 containing pRS313-IRE1 carrying the K1058A cytosolic-domain point mutation by using a primer set shown in Table S4 and XbaI/NotI-digested pRS313-IRE1. The bZIP mutant version of pRS313-IRE1 was described previously^12^. W303-ire1Δ transformed with an empty vector pRS313^32^ were used as *ire1Δ* cells. In order to obtain the *IRE1*+ *hac1Δ* cells that appear in Figs. S3b and 7f, W303-ire1Δ cells carrying pRS313-IRE1 were further transformed with the *hac1::KanMX4* gene-disruption module which had been PCR amplified from a EUROSCARF gene disruption-library strain. For cellular expression of eroGFP and mitochondira-localized GFP, we used plasmids pPM28 and pYX142-mtGFP respectively^21,33^.

### Chemicals

DTT (Nakalai Tesque), tunicamycin (Sigma Aldrich), antimycin A (Sigma Aldrich) and NAC (Sigma Aldrich) were respectively prepared as stock solutions of 1 M in water, 2 mg/ml in dimethyl sulfoxide (DMSO), 20 mM in DMSO and 0.5 M in water, and stored at −30 °C. Hydrogen peroxide was obtained as a 35% water solution (Nakalai Tesque). For chemical-stress imposition, DTT, tunicamycin or hydrogen peroxide was added into cultures during the exponential and fermentative growing phase.

### RNA and DNA analyses

The hot phenol method was used for extraction of total RNA from yeast cells^34^. As described previously^12^, the RNA samples were then used for reverse transcriptase (RT)-PCR amplification of the *HAC1* transcripts using a polyA RT primer and *HAC1*-specific PCR primers (Table S5). The PCR products were then analyzed by 2% agarose-gel electrophoresis (TBE buffer), and ethidium bromide-fluorescent images of the resulting gels were pictured with a GelDoc XR+ imaging system (BioRad) and are presented in Figs 1a,c, S2 and S4b. Alternatively, the gel images were used for image quantification, the results of which were used to calculate the values of the *HAC1* mRNA-splicing efficiency using the formula [100 × (band intensity of *HAC1*^i^)/{(band intensity of *HAC1*^i^) + (band intensity of *HAC1*^u^)}], where *HAC1*^i^ and *HAC1*^u^ respectively are the spliced and unspliced forms of the *HAC1* mRNA.

In order to remove residual genomic DNA from the total RNA samples before real-time quantitative RT-PCR (qRT-PCR) analysis and high-throughput RNA-seq analysis, samples were treated with recombinant DNase I (RNase-free; Takara) as per the manufacturer’s instruction. For the qRT-PCR analysis, the resulting total RNA samples were subjected to the RT reaction as per the RT-PCR amplification of the *HAC1* transcripts. The RT-reaction products were analyzed with SYBR Premix Ex Taq™ II (Tli RNaseH Plus; Takara) on a real-time PCR machine LightCycler 96 (Roche). The quantitative PCR (qPCR) conditions were as follows; pre-incubation for 95 °C 5 min and 3-step cycle amplification for 95 °C 10 sec/60 °C 10 sec/72 °C 10 sec. Primer sets used in this analysis are listed in Table S5. The *TAF10* primer sets served as the internal control.

Genomic DNA was extracted from yeast cells using GenTLE yeast DNA extraction kit (Takara) and analyzed by qPCR.

Preparation of next generation sequencing (NGS) libraries and NGS were performed at the Eurofinesgenomics K.K. (Tokyo) under the following conditions: sequencer: HiSeq2500 (Illumina), read length: 125 bases of paired-end, software: bclfastq2 software v1.8.4 (Illumina). The NGS data were deposited in ArrayExpress (https://www.ebi.ac.uk/arrayexpress/; accession E-MTAB-6923). After NGS analysis, fastq files were processed for trimming, filtering and mapping with CLC genomic work bench v10.1(Qiagen) under the default setting. The processed NGS data were mapped on *Saccharomyces cerevisiae* S288C genome version R64-1-1. Based on the mapped data, TPM as gene expression values were calculated with CLC genomic work bench v10.1.

### Protein analysis

After harvest by centrifugation at 1,600 × g for 1 min, 5.0 OD_600_ cells were disrupted by agitation with glass beads (425–600 µm) in 100 µl of the lysis buffer containing 50 mM Tris-Cl (pH7.9), 5 mM ethylenediaminetetraacetic acid and 1% Triton X-100 and protease inhibitors (2 mM phenylmethylsulfonyl fluoride, 100 µg/ml leupeptin, 100 µg/ml aprotinin, 20 µg/ml pepstatin A and Calbiochem Protease Inhibitor cocktail Set III (X100 dilution)), and were clarified by centrifugation at 8,000 × g for 1 min. For the BiP sedimentation assay^19^, crude cell lysates were sequentially centrifuged at 700 × g for 3 min and at 8,000 × g for 20 min, and the pellet fractions obtained from the second centrifugation were further analyzed as the “pellet” samples in the experiment shown in Fig. S6a. The resulting samples were subjected to standard SDS-DTT polyacrylamide gel electrophoresis (PAGE), which is followed by Western blotting with 12CA5 anti-HA antibody (Roche; 1:2,000 dilution), anti-PGK1 antibody (1:2,000 dilution) or polyclonal anti-yeast BiP antiserum (ref.^35^, 1:3,000 dilution). Mouse monoclonal antibody 6G9E6BC4 (Abcam; 1:1,000 dilution) was used for Western-blot detection of Por1. After treatment with the horseradish peroxidase-conjugated secondary antibodies and enhanced chemiluminescence (ECL) reagent (ECL prime, GE healthcare), the blot membranes were subjected to image analysis in the luminescence imager LAS4000 (Fuji Film).

### Microscopic analysis

In order to obtain fluorescent images of eroGFP, cells transformed with pPM28 were observed using a Keyence BZ-9000E microscope carrying a CFI Plan Apo λ100xH objective lens (Nikon). We used the fluorescence filter set OP-79301 for excitation with blue light (1.0 sec exposure) and a custom-made fluorescence filter set (excitation wavelength 395/25, dichroic mirror wavelength 495, emission 510/20) for excitation with UV/violet light (1.3 sec exposure). The ratio of the signal intensities under blue-light excitation and that under UV/violet-light excitation of each cell was obtained using the image processing software ImageJ (https://imagej.nih.gov/ij/). At least 30 cells were analyzed for each culture sample.

In order to obtain fluorescent images of mitochondria-located GFP, cells transformed with pYX142-mtGFP were observed using a confocal laser scanning microscope LSM700 (Zeiss) carrying a Plan-Apochromat 100x/1.40 objective lens (Zeiss) under a pinhole of 0.74 airy units (argon laser at 100% output) and in the EGFP smart-setup and auto-expose mode. We obtained a confocal fluorescent image (512 × 512 pixels, zoom 2.4) after focusing on the center of a cell, which was then binarized using Image J (default setting) for measuring mitochondria area. In the experiment shown in Figs 8c and S10C, approximately 100 cells (the mother cell in the case of a budded cell) were analyzed for their mitochondria area.

### Flow cytometry

After addition of DCFH-DA (5.0 µg/ml final concentration), DHE (2.5 µg/ml final concentration) or MitoSOX Red (Thermo Fisher Scientific, 5.0 µg/ml final concentration) into cultures and further incubation for 30 min, cells were analyzed on a Accuri C6 flow cytometer (BD Biosciences). The “DCFH-DA-derived fluorescence” value was calculated by subtraction of the mean fluorescence value (the FL1 (530/30 nm) channel) of unstained cells (20,000 cells) from that of DCFH-DA stained cells (20,000 cells). DHE-derived fluorescence and MitoSOX Red-derived fluorescence were similarly detected through the FL2 (585/40 nm) channel and calculated.

### Oxygen consumption monitoring

MitoXpress Xtra (Luxcel Biosciences) contains a fluorophore (Ex_max_: 380 mM and Em_max_: 650 nm), the fluorescence of which is quenched by dissolved oxygen. After being aerated for 1-min by vortexing at top-speed, 300 µl of post-diauxic-shift culture was quickly mixed with 25 µl of the MitoXpress Xtra solution and put into a quartz cell, which was then sealed with mineral oil before measurement using a F-7000 fluorescence spectrophotometer (Hitachi; excitation 380 nm (slit width: 5 nm); emission 650 nm (slit width: 5 nm) and 675 nm (for background, slit width: 5 nm)). Then increment of the fluorescence intensity was monitored for every 15 sec for calculation of the oxygen consumption rate.

### Statistics

In order to express quantified *HAC1*-mRNA splicing, culture optical density, Western blotting, RT-qPCR, qPCR and oxygen consumption data throughout this report, we obtained the average values with standard deviations from results from multiple independent transformant clones carrying the same plasmid (pRS313, pRS313-IRE1 or its mutants), which are presented in the Figures and tables.

To evaluate statistical differences, we performed the paired (Figs 4d, 5b,d and 6a,b) or unpaired two-tailed Student’s t-test, the results of which are expressed as “p value”.

## Supplementary information

Supplemental Figures and Tables

Table S3
