# Supplementary material for: The unfolded protein response alongside the diauxic shift of yeast cells and its involvement in mitochondria enlargement
Source: Sci Rep. 2019 Sep 4;9:12780. doi: 10.1038/s41598-019-49146-5 (PMC6726593; doi:10.1038/s41598-019-49146-5)
Supplement: Supplementary file 1 — Supplemental Figures and Tables [file 41598_2019_49146_MOESM1_ESM.pdf]

Supplementary Information

The unfolded protein response alongside the diauxic shift of yeast cells and its involvement in mitochondria enlargement

Duc Minh Tran, Yuki Ishiwata-Kimata, Thanh Chi Mai, Minoru Kubo and Yukio Kimata*

Affiliations

Graduate School of Science and Technology, Nara Institute of Science and Technology, 8916-5 Takayama, Ikoma, Nara 630-0192, Japan
Duc Minh Tran, Yuki Ishiwata-Kimata, Thanh Chi Mai and Yukio Kimata

Institute of Biotechnology, Vietnam Academy of Science and Technology, 18 Hoang Quoc Viet road, Cau Giay, Ha Noi, Viet Nam
Duc Minh Tran

Institute for Research Initiatives, Nara Institute of Science and Technology, 8916-5 Takayama, Ikoma, Nara 630-0192, Japan
Minoru Kubo

*Corresponding author:
Yukio Kimata
kimata@bs.naist.jp
tel: +81-80-6109-8974

Figure S1

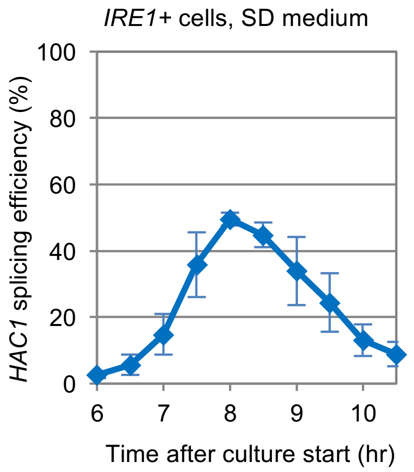

Figure S1: Detailed time-course profiling of the transient *HAC1* mRNA splicing upon diauxic shift. The same experiment as shown in Fig. 1b was performed with 30-min sampling intervals.

Figure S2

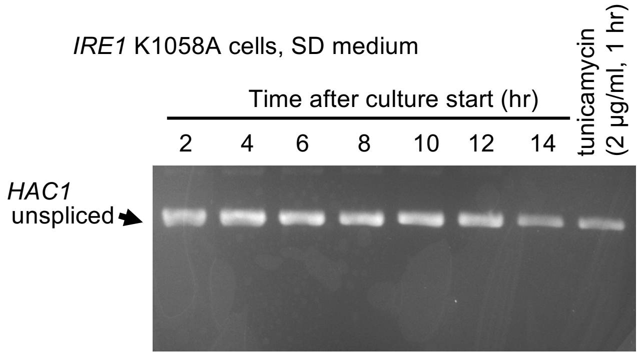

Figure S2: The K1058A mutation of *IRE1* abolishes the *HAC1* mRNA splicing. The same experiment as described in Fig. 1A was performed using cells carrying the K1058A mutation on the *IRE1* gene.

Figure S3

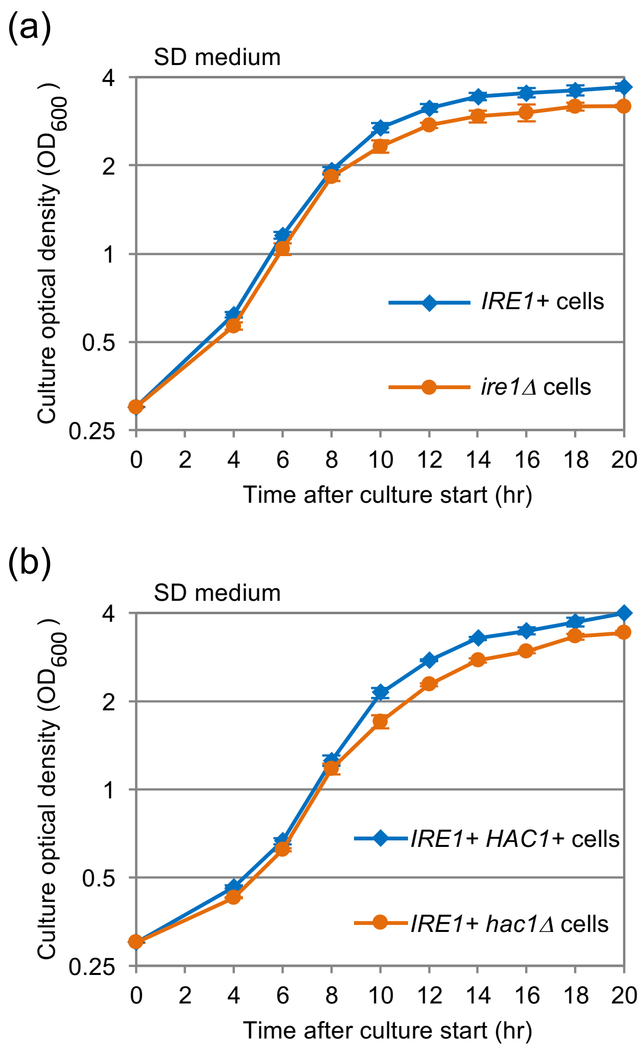

Figure S3: The Ire1-*HAC1* pathway contributes to cell growth upon diauxic shift. Cells were grown in SD medium, and optical density of cultures at the indicated time points was monitored.

Figure S4

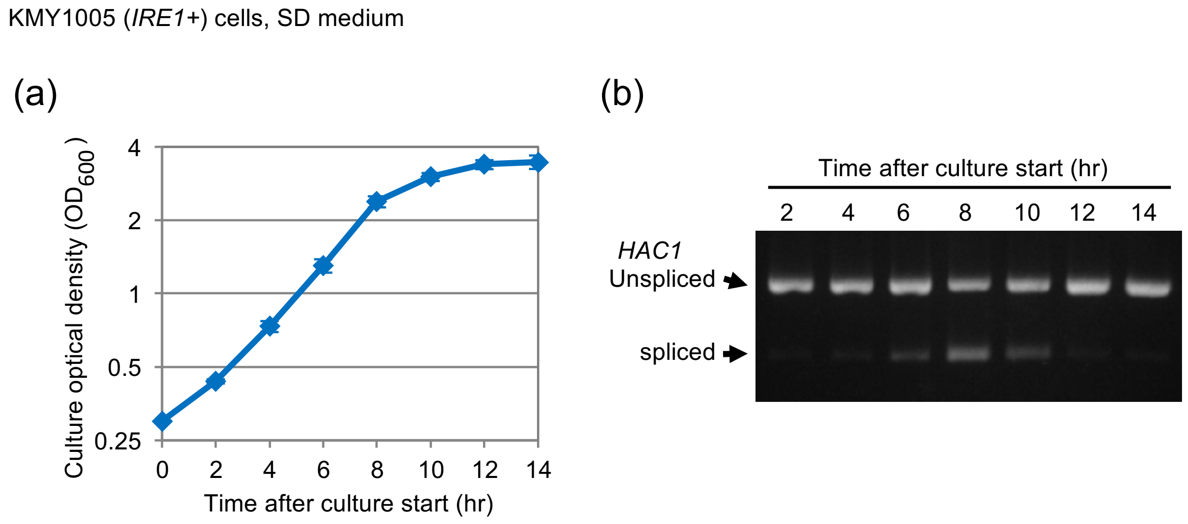

Figure S4: Transient induction of the *HAC1*-mRNA splicing upon diauxic shift in yeast KMY1005 strain. KMY1005 cells were cultured in SD medium and checked for cellular growth (a) and *HAC1*-mRNA splicing (b).

Figure S5

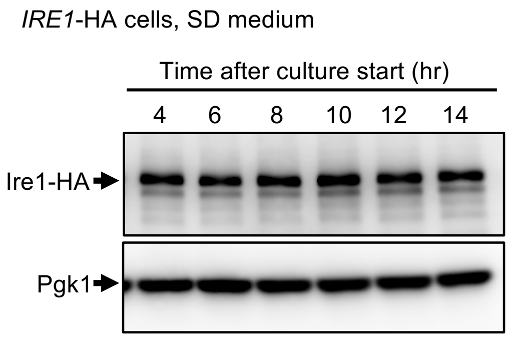

Figure S5: Time-course change of the cellular Ire1 level. Cells carrying Ire1-HA were cultured in SD medium for the indicated durations. Their lysates (equivalent to 0.1 OD_{600} cells) were subjected to anti-HA Western-blot analysis. Anti-Pgk1 Western blot serves as a loading control.

Figure S6

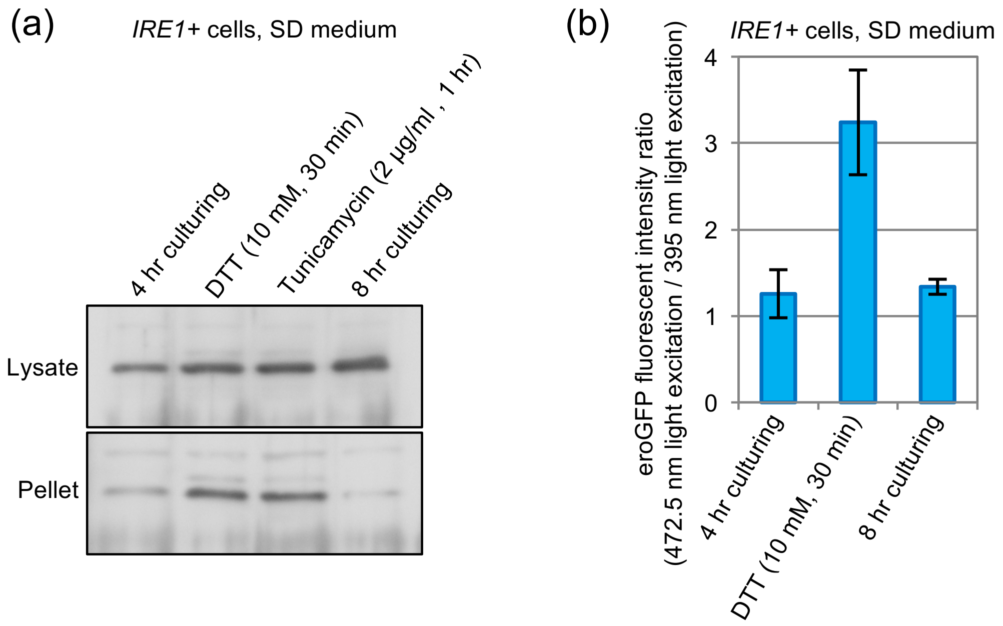

Figure S6: ER protein-folding status is not impaired upon diauxic shift. (a) After being cultured for the indicated durations or stressed by DTT or tunicamycin, *IRE1*+ cells were lysed and subjected to high-speed centrifugation as described in the Materials and Methods section. The total cell lysates (equivalent to 0.05 OD_{600} cells) or the pellet fractions (equivalent to 1.0 OD_{600} cells) were subjected to anti-BiP Western-blot analysis. (b) After being cultured for the indicated durations or stressed by DTT, *IRE1*+ cells producing eroGFP were illuminated by UV/violet light or blue light for detection of green fluorescent signals.

Figure S7

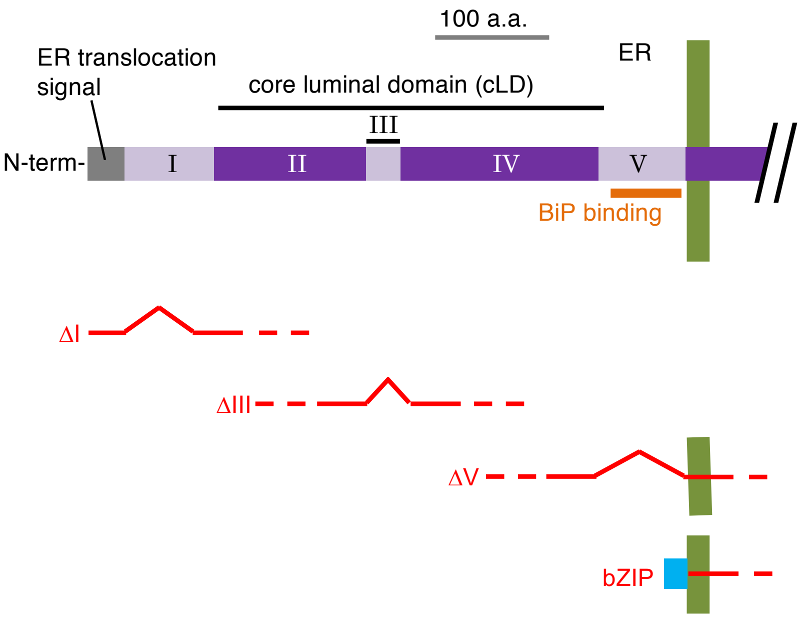

Figure S7: Structure of the luminal domain of yeast Ire1 and its mutants. Segmentation of the luminal domain of yeast Ire1 into Subregions I to V is described in Ref 5. Subregions II and IV form one tightly folded module, namely the core luminal domain, which is responsible for unfolded-protein sensing and self-association (12). Meanwhile, Subregions I and V are loosely folded and play suppressive roles in Ire1 regulation. The ΔI, and ΔIII and ΔV mutations are deletions of a.a 32-91, a.a. 253-272 and a.a. 463-524 of yeast Ire1, respectively (7).

Figure S8

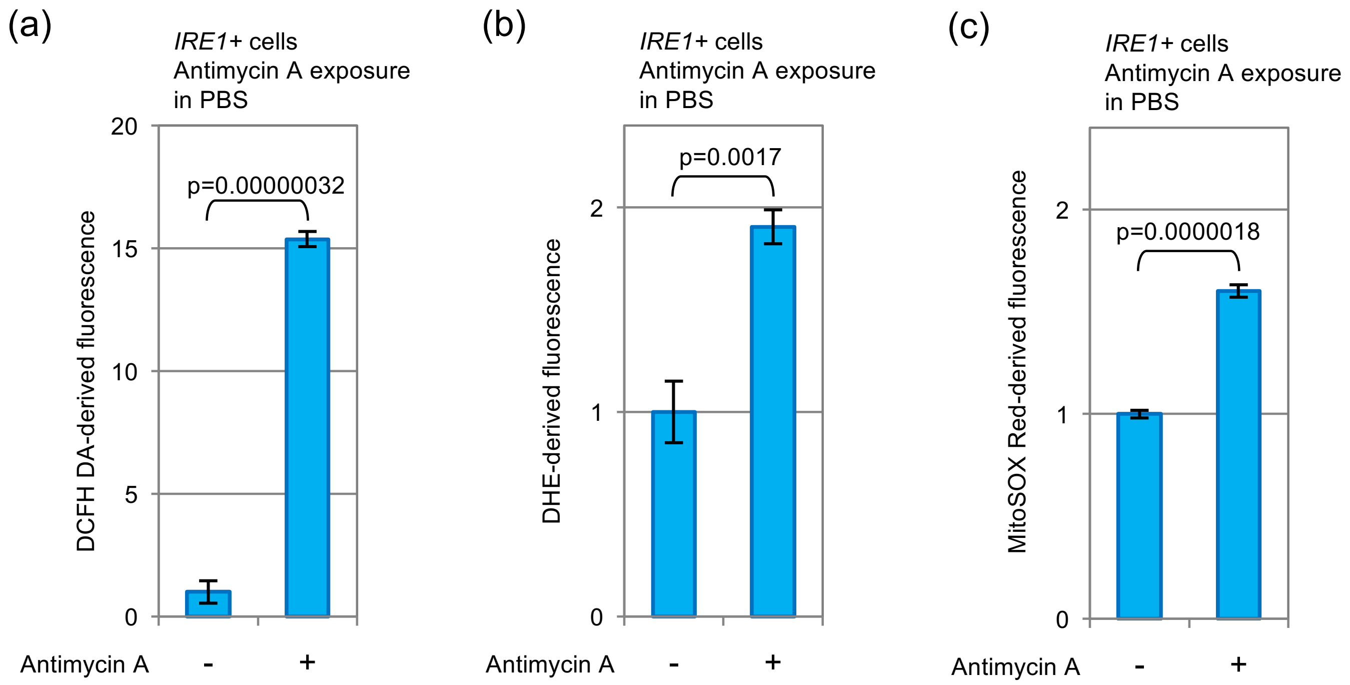

Figure S8: Effect of antimycin A exposure on the ROS level in PBS-suspended cells. After being cultured in SD medium and harvested, cells were suspended in PBS and incubated with or without 20 μM antimycin A in the presence of the indicated ROS-indicator dyes for 30 min, and were then analyzed by flowcytometry. The values are normalized against those of the left bars (antimycin A –), which are set at 1.00.

Figure S9

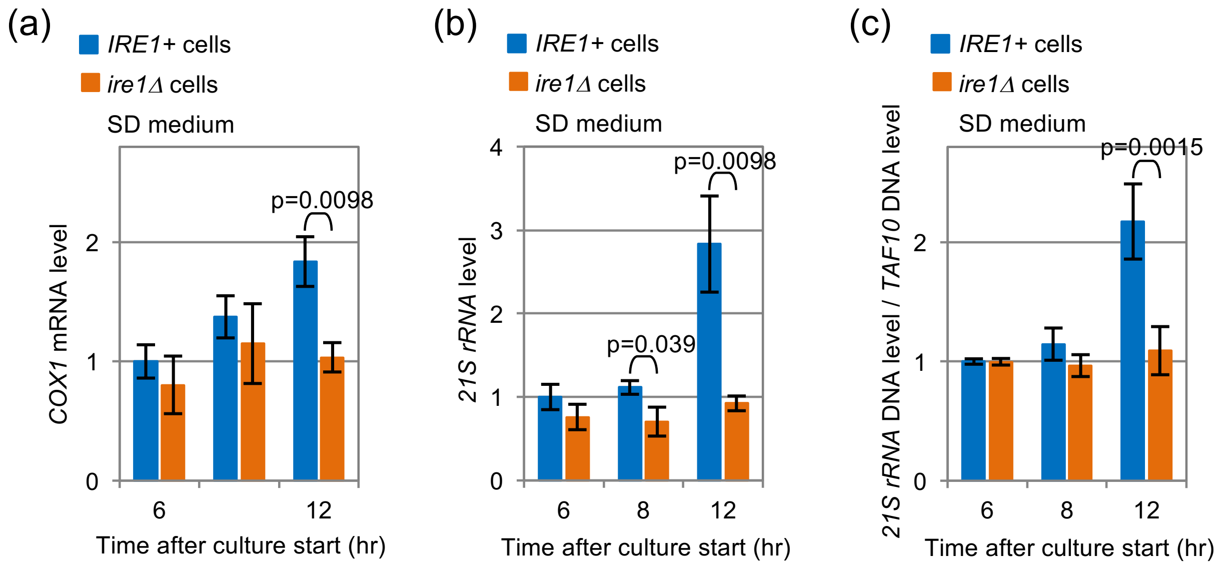

Figure S9: Change of the mitochondrial RNA and DNA levels in *IRE1*+ cells and *ire1Δ* cells upon their long-time culturing. RNA or DNA samples prepared in the experiments shown in Fig. 7 were subjected to RT-qPCR or genomic qPCR analyses using mitochondrial genome-specific oligonucleotide primers.

Figure S10

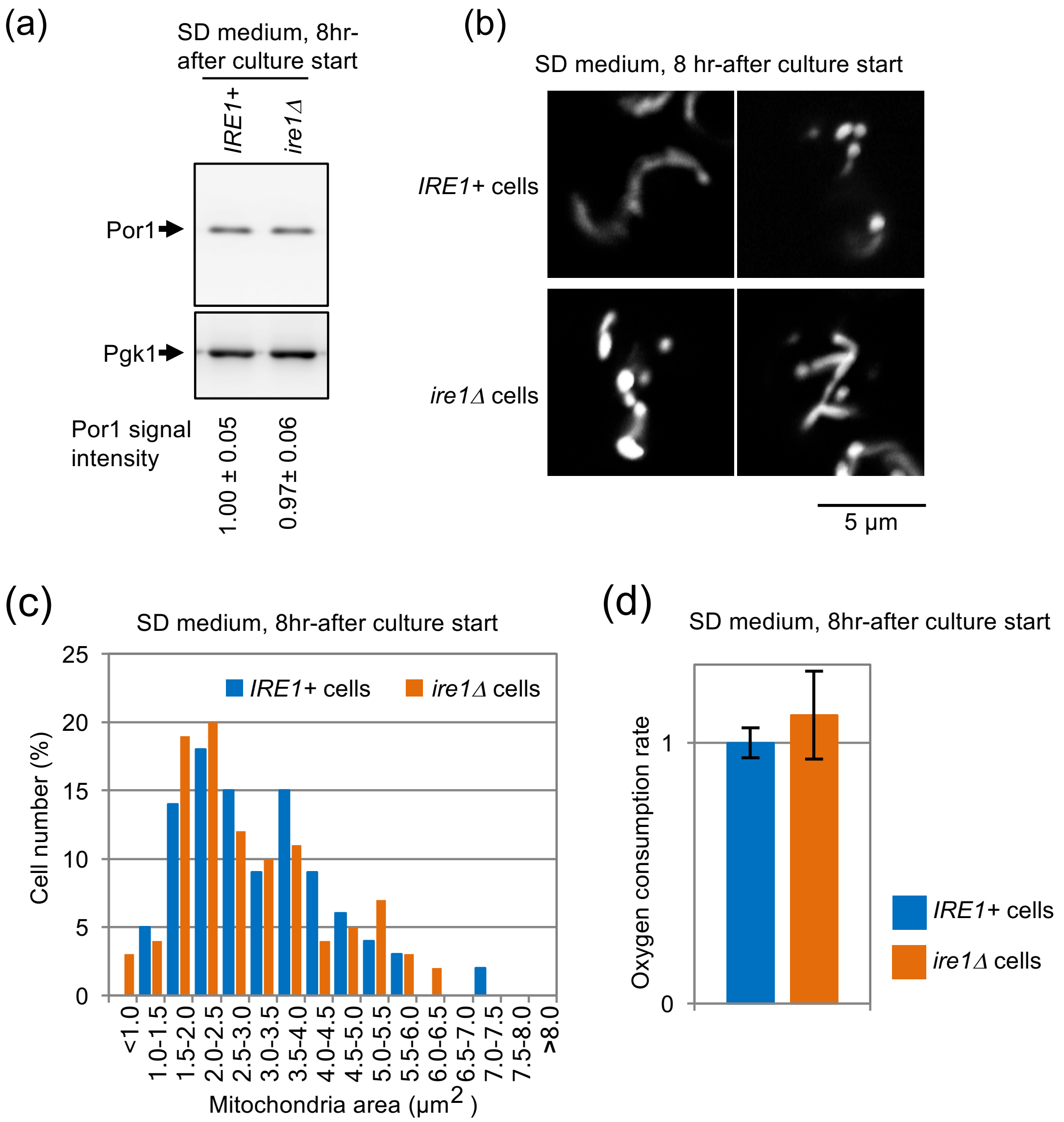

Figure S10: Comparison of mitochondria-related phenotypes between 8hr-cultiured *IRE1*+ and *ire1Δ* cells. After being cultured for 8 hr in SD medium, *IRE1*+ cells and *ire1Δ* cells were subjected to the same analyses as done in Fig. 8. In panel c, the mitochondria size distribution of *IRE1*+ cells was not statistically different from that of *ire1Δ* cells (p=0.29)

Figure S11

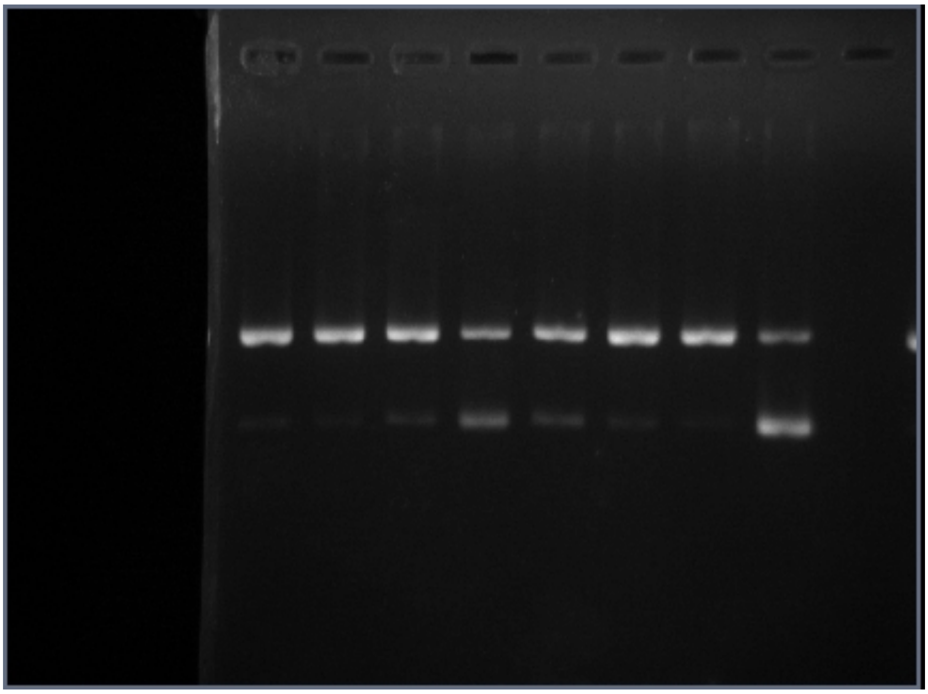

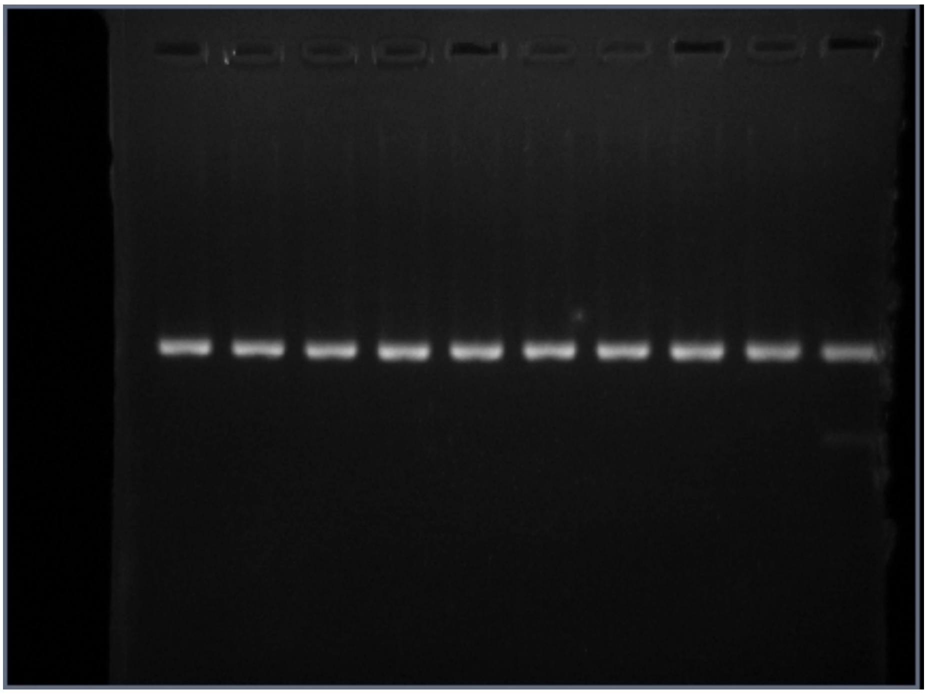

Figure S11: Uncropped gel images for Figs 1a and d.

Figure S12

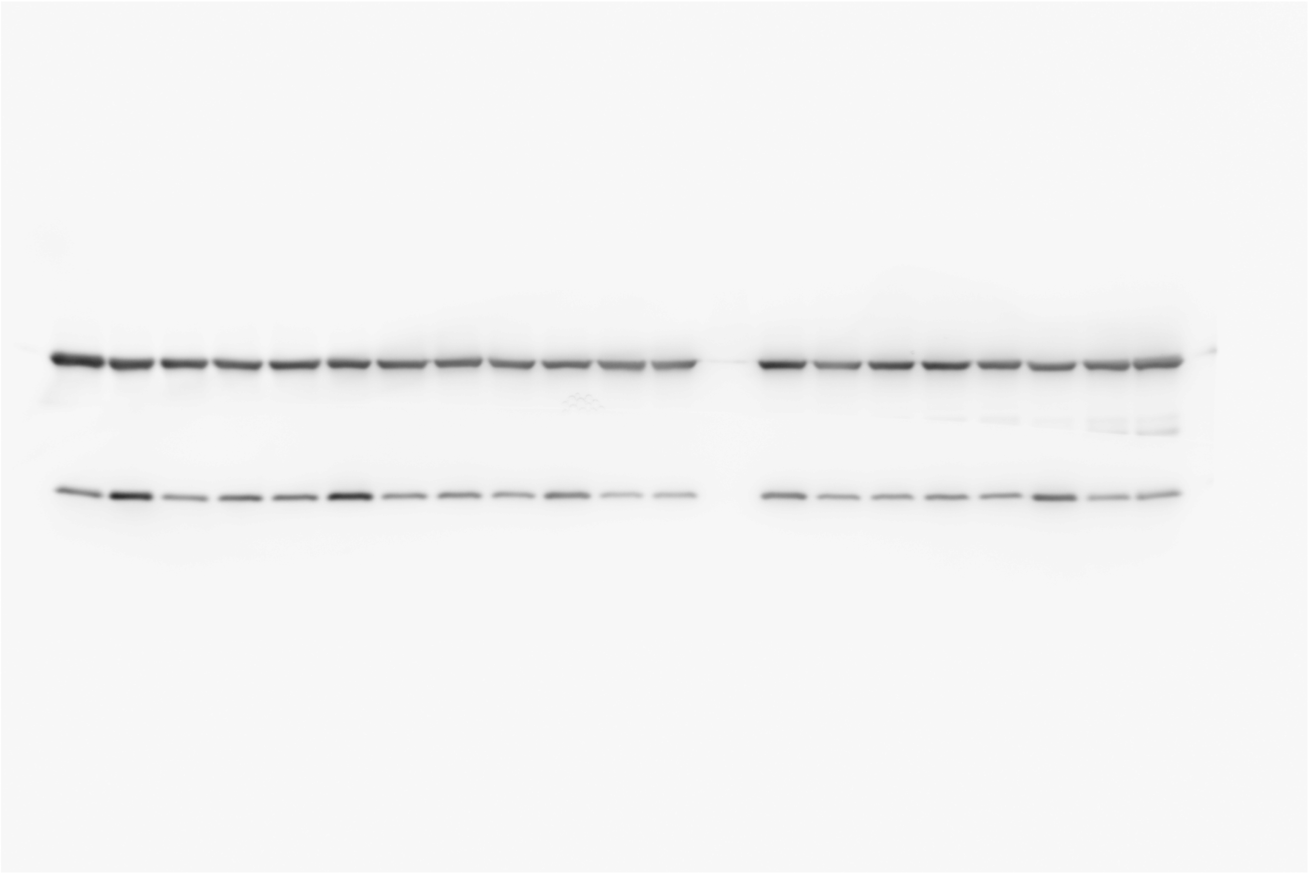

Figure S12: Uncropped blot image for Fig. 8a.

Table S1 Slopes of the growth charts shown in this paper

Growth chart	Fig. 1C	Fig. 1F	Fig. 3A	Fig. 3A	Fig. 3C	Fig. 3E	Fig. 3E	Fig. 3E	Fig. 3E	Fig. 4A	Fig. 4C	Fig. 4C	Fig. 5A	Fig. 5A	Fig. 6A	Fig. 6A	Fig. S4A
IRE1 genotype	WT	WT	WT	ΔIII	bZIP	WT	ΔI	ΔV	ΔIΔV	ρ0	WT	WT	WT	WT	WT	WT	WT KMY1005
medium	SD	YPD	SD	SD	SD	SD	SD	SD	SD	SD	SD	SD+antimycinA	SD	SD+NAC	SCG	SCG	SD
HAC1 splicing chart	Fig. 1B	Fig. 1E	Fig. 3B	Fig. 3B	Fig. 3D	Fig. 3F	Fig. 3F	Fig. 3F	Fig. 3F	Fig. 4C	Fig. 4D	Fig. 4D	Fig. 5B	Fig. 5B	Fig. 6B	Fig. 6B	Fig. S5B
Fold change of the culture density during 2-hr culturing																	
0 hr to 2 hr	1.47	1.42	1.49	1.64	1.38	1.07	1.20	1.15	1.11	1.25	1.45	1.52	1.43	1.28	2.27	2.22	1.46
2 hr to 4 hr	1.62	2.21	1.68	1.68	1.50	1.76	1.85	1.80	1.72	1.31	1.70	1.57	1.63	1.32	1.14	0.93	1.68
4 hr to 6 hr	1.80	2.28	1.95	1.92	1.73	1.83	1.79	1.72	1.63	1.18	2.02	1.91	1.88	1.54	1.45	1.22	1.77
6 hr to 8 hr	1.72	1.26	1.72	1.69	1.49	1.77	1.67	1.97	1.65	1.76	1.78	1.89	1.47	1.37			1.83
8 hr to 10 hr	1.26	1.10	1.27	1.22	1.48	1.36	1.28	1.15	1.50	1.38	1.26	1.30	1.46	1.53			1.26
10 hr to 12 hr	1.16	1.06	1.09	1.06	1.09	1.14	1.08	1.14	1.17	1.49	1.06	1.02	1.16	1.20			1.12
12 hr to 14 hr	1.08	1.11	1.06	1.06	1.08	1.05	1.09	1.05	1.03	1.13	1.04	1.08	1.06	1.15			1.02

The slopes of the lines in the indicated figures are indicated.

Table S2 Comparison of growth rate between two genotypes

Growth chart	Fig. S2A								
Genotype	IRE1+				ire1Δ				
Clone number	1	2	3	Average	1	2	3	Average	t.test
Fold change of the culture density during 2-hr culturing									
4 hr to 6 hr	1.81	1.88	1.91	1.87	1.92	1.76	1.82	1.83	0.56
6 hr to 8 hr	1.62	1.75	1.61	1.66	1.76	1.92	1.63	1.77	0.32
8 hr to 10 hr	1.39	1.36	1.45	1.40	1.24	1.32	1.25	1.27	0.02
10 hr to 12 hr	1.18	1.21	1.12	1.17	1.21	1.11	1.23	1.18	0.78
12 hr to 14 hr	1.10	1.06	1.13	1.09	1.11	1.07	1.04	1.07	0.47
14 hr to 16 hr	1.02	1.05	1.03	1.03	1.06	1.02	1.01	1.03	0.95
16 hr to 18 hr	1.08	0.95	1.03	1.02	0.96	1.11	1.09	1.05	0.62
18 hr to 20 hr	0.98	1.10	1.00	1.03	1.02	0.97	1.02	1.00	0.58

Growth chart	Fig. S2B								
Genotype	IRE1+HAC1+				IRE1+hac1Δ				
Clone number	1	2	3	Average	1	2	3	Average	t.test
Fold change of the culture density during culturing for 2 hours									
4 hr to 6 hr	1.46	1.46	1.37	1.43	1.46	1.47	1.44	1.46	0.44
6 hr to 8 hr	1.78	1.99	1.89	1.89	1.90	1.95	1.81	1.89	0.99
8 hr to 10 hr	1.83	1.63	1.66	1.70	1.41	1.49	1.45	1.45	0.02
10 hr to 12 hr	1.25	1.29	1.35	1.29	1.33	1.25	1.44	1.34	0.51
12 hr to 14 hr	1.19	1.16	1.21	1.19	1.25	1.22	1.17	1.21	0.43
14 hr to 16 hr	1.02	1.08	1.09	1.06	1.07	1.05	1.09	1.07	0.77
16 hr to 18 hr	1.06	1.08	1.07	1.07	1.11	1.17	1.10	1.13	0.07
18 hr to 20 hr	1.13	1.06	1.04	1.07	1.01	1.02	1.07	1.03	0.26

In Fig. S3A and S3B, three independent clones with the same genotype were monitored for culture optical density. We thus calculated its fold change during 2-hr culturing, and evaluated if the resulting values from two genotypes are statistically different as determined by Student's two-tailed t test. Yellow shaded rows: $p<0.05$.

Table S4 IRE1 mutation primers used for overlap PCR

For luminal domain mutation

Internal mutation primers

Mutation	Orientation	Sequence
ΔI	Forward	TGCTCAATCCCATTGTCGTCTCGCCGTGCTAACAAAAAAGGACGTAGG
	Reverse	CCTACGTCCTTTTTTGTTAGCACGGCGAGACGACAATGGGATTGAGCA
ΔIII	Forward	CAGCGTTCGGACCTGGTTCAAAAGAATCTGAAAATATGATTGTAATAGGC
	Reverse	GCCTATTACAATCATATTTTCAGATTCTTTTGAACCAGGTCCGAACGCTG
ΔV	Forward	CACCTTTATGAAAACTATGAAAAACAAAATTCTTTGCTACTGAAGTTTG
	Reverse	CAAACTTCAGTAGCAAAGAATTTTGTTTTTCATAGTTTTCATAAAGGTG

External primers

Orientation	Sequence
Forward	CCATTATCACTTTTCTCCATATCA
Reverse	CCTTGAAAACTTCCCTGAAAAACT

For cytosolic domain mutation

Internal mutation primers

Mutation	Orientation	Sequence
K1058A	Forward	GAGCACTTAGGAATGCATATCATCAT
	Reverse	ATGATGATATGCATTCCTAAGTGCTC

External primers

Orientation	Sequence
Forward	CGATGATGCTGATGAAGATGATGA
Reverse	ACGTTGTAAAACGACGGCCAGTGA

Table S5: Oligonucleotide primers for qPCR or RT-qPCR.

Treget gene		Sequence
HAC1	Forward	TACAGGGATTTCCAGAGCACG
	Reverse	TGAAGTGATGAAGAAATCATTCAATTC
KAR2	Forward	TCTGAAGGTGTCTGCCACAG
	Reverse	TTAGTGATGGTGATAGATTCGGATT
PDI1	Forward	TACGAAGAAGCCCAGGAAAA
	Reverse	GTCAGCCAATTCAGCGTCA
TSA1	Forward	GTTCATCATCGACCCAAAGG
	Reverse	GTCAACGTTTCTACCGACTGG
OLI1	Forward	GCAGGTATTGGTATTGCTATCG
	Reverse	GCTTCTGATAAGGCGAAACC
COX1	Forward	TCCAACAGGAATTAAAATTTTCTCA
	Reverse	GCAATTGCATATAACATAGGTAGTGC
21S rRNA	Forward	AAATTGAAATCGTAGTGAAGATGCT
	Reverse	AAAGCTGCATAGGGTCTTTCC
TAF10	Forward	ATATTCCAGGATCAGGTCTTCCGTAGC
	Reverse	GTAGTCTTCTCATTCTGTTGATGTTGTTGTTG
